# Modulation of structure, functionality, allergenicity and *in vitro* digestibility of ovalbumin through cold plasma-induced conjugation with rosemary-derived phenolic acids

**DOI:** 10.1016/j.fochx.2026.104409

**Published:** 2026-09-06

**Authors:** Zhi-Wei Liu, Xiang-Xiang Xu, Zi-Jing Yin, Waqar Ahmed, Marwa Ezz El-Din Ibrahim, Rana Muhammad Aadil, Hong-Ying Guo

**Affiliations:** a College of Food Science and Technology, Hunan Agricultural University, Changsha 410128, China; b National Institute of Food Science and Technology, University of Agriculture, Faisalabad 38000, Pakistan; c Department of Food and Nutrition Sciences, College of Agricultural and Food Sciences, King Faisal University, Al-Ahsa 31982, Saudi Arabia

**Keywords:** Ovalbumin, Cold plasma, Rosemary-derived phenolic acids, Allergenicity, Digestibility

## Abstract

Cold plasma (CP)-induced covalent conjugation between ovalbumin (OVA) and three rosemary-derived phenolic acids (RDPAs), namely chlorogenic acid (CGA), caffeic acid (CA), and rosmarinic acid (RA), was investigated to elucidate how phenolic acid molecular architecture governs the structure, functionality, *in vitro* digestibility, and allergenicity of OVA. After 45 s of CP treatment (40 kV, 13 kHz), the covalent grafting efficiency of OVA-RDPAs conjugates showed a clear structure dependence, following the order OVA-RA (86.55 ± 2.39 μmol/g) > OVA-CA (79.27 ± 1.62 μmol/g) > OVA-CGA (53.07 ± 2.16 μmol/g). This distinct hierarchy is attributed to RA's dual aromatic rings and four evenly distributed hydroxyl groups, which provide more accessible reactive sites, in contrast to the substantial steric hindrance imposed by CGA's bulky cyclohexane moiety. Multi-spectroscopic and SDS-PAGE analyses revealed that RDPAs grafting induced pronounced conformational unfolding and intermolecular cross-linking in OVA. Consequently, these structure-driven changes translated into remarkably augmented emulsifying and antioxidant properties and lower allergenicity of OVA. Among the conjugates, OVA-RA exhibited superior improvements, achieving 5.1-fold and 14.6-fold increases in DPPH and ABTS radical scavenging capacities, respectively. Notably, RDPAs incorporation effectively masked antigenic epitopes, and OVA-RA exhibited the greatest reduction in IgE-binding capacity (65.81 ± 0.74%). Furthermore, covalent grafting of RDPAs, particularly RA, effectively promoted gastrointestinal digestive trajectories of OVA, and the resulting digests substantially reduced IgE-binding affinity. These findings suggest that CP-mediated covalent RDPAs conjugation is a mild and effective, structure-guided approach that holds potential for the development of egg protein ingredients with enhanced functionality and reduced allergenicity.

## Highlights

• CP enabled rapid covalent conjugation of OVA with RDPAs within 45 s.
• RDPAs structure governed grafting efficiency, with order OVA-RA > OVA-CA > OVA-CGA.
• OVA-RA enhanced antioxidant activity, with 5.1-fold and 14.6-fold increases in DPPH and ABTS.
• Covalent grafting of RDPAs reduced OVA allergenicity and improved its digestive behavior.

## 1 Introduction

OVA is routinely incorporated into a broad spectrum of processed foods, ranging from baked goods and emulsified condiments to high-protein specialty formulations (Rostamabadi et al., 2023). However, despite all these applications, the following drawbacks have been identified regarding native OVA: low antioxidant property, moderate emulsification stability during processing operations, and a clinical problem of egg allergy that affects a sizable portion of the world's population (Razi et al., 2023; Vapor et al., 2022). Thus, addressing these limitations while maintain OVA functionality remains an ongoing objective. Among the available modification strategies, covalent conjugation between proteins and polyphenols has attracted increasing attention as an effective approach to enhance protein functionality (Man et al., 2024). Polyphenols can be oxidized to generate reactive quinone intermediates, which readily react with nucleophilic residues in proteins, such as lysine, cysteine, and tyrosine, to form stable covalent conjugates. To promote protein–polyphenol conjugation, several methods have been proposed, including alkaline oxidation, enzymatic catalysis, and free radical-mediated reactions (Shang et al., 2024; Wu et al., 2024). Under alkaline conditions, phenolic hydroxyl groups are oxidized into quinones that subsequently react with protein side chains (Chen et al., 2026). Enzymatic approaches employ oxidases such as laccase or tyrosinase to catalyze phenolic oxidation and cross-linking (Wu et al., 2024). Free radical-mediated methods, often initiated by chemical oxidants or ultrasound-assisted systems, generate reactive radicals that accelerate conjugate formation (Wang et al., 2024). Despite their effectiveness, these methods often involve harsh reaction conditions, high processing costs, or uncontrolled oxidative reactions, which may adversely affect protein structure and nutritional quality. Therefore, the development of efficient and mild techniques for protein–polyphenol conjugation remains highly desirable.

As a novel non-thermal processing method, CP has gained widespread recognition for its versatile applications in food science (Deng et al., 2025). In CP, the combination of reactive oxygen species and nitrogen species, charged particles and ultraviolet photons produces a chemical reaction environment that can induce oxidative modification and free radical-mediated covalent coupling between proteins and phenolic acids without the use of extreme temperatures. (Yawut et al., 2024). In protein-polyphenol systems, CP rapidly oxidizes phenolic hydroxyls to semiquinone/quinone intermediates. At the same time, it activates protein side chains for nucleophilic attack, allowing simultaneous covalent coupling of both substrates (Song et al., 2024). When compared to commonly used free radical, alkaline, and enzymatic methods, CP is advantageous as it utilizes significantly shorter processing times, operates at room temperature, does not produce chemical additives and has much better yields of conjugation. Previous studies in our laboratory showed that the CP treatment of tea catechins (EC, EGC, EGCG) in less than 45 s successfully covalently grafted them to food allergen proteins. The bioconjugates showed distorted structures, entrapment of IgG/IgE-accessible epitopes, reduced antibody activity and binding, and increased antioxidant activity and interfacial properties (Z.-W. Liu, et al., 2025). However, there is a lack of systematic studies on CP-assisted conjugation of OVA and plant-derived phenolic acids.

Three distinct polyphenols like CGA, CA, and RA can be derived from rosemary (*Rosmarinus officinalis*), a popular culinary and nutraceutical herb that is widely employed for their antioxidant and antimicrobial polyphenols in food and medicine (Noor et al., 2022). All three phenolic acids have been previously reported to exhibit a variety of biological activities. While CGA, CA, and RA all possess two phenolic hydroxyls on a benzene ring (a catechol group), the side chains of these phenolic acids have very different structures. CGA is an ester of caffeic acid and quinic acid with a large substituent that is a cyclohexane polyol. CA only has one ring and is a simple trans-hydroxycinnamic acid with a catechol group, and RA is a dimeric phenylpropanoid compound containing two catechol groups, each bearing two phenolic hydroxyl groups on its benzene ring. (Chaowuttikul et al., 2020). Thus, their different structures will determine their oxidation rates, affinities to quinones, and conjugation efficiencies when grafting onto OVA. While the binding between these RDPAs and some proteins has been reported before, the vast majority of these studies were on the non-covalent binding or involved synthesizing common adducts other than utilizing the unique radical formation (Lu et al., 2022; Shang et al., 2024). C. Liu, et al. (2025) recently demonstrated CP-induced structure-dependent conjugation efficiency for monomeric catechins (EC, EGC, EGCG). However, the efficiency of CP for OVA–RDPAs covalent conjugation and the influence of these distinct phenolic structures on conjugation efficiency and the final functionalities have not been systematically investigated.

Therefore, the present study aimed to investigate CP-induced covalent conjugation between OVA and RDPAs, namely CGA, CA, and RA. Conjugation efficiency and structural changes in OVA were characterized by SDS-PAGE, mutil-spectroscopic analysis. Potential binding sites was identified by LC-MS/MS. In addition, the effects of conjugation on antioxidant activity, emulsifying properties, *in vitro* digestibility, and antigenicity were systematically evaluated. This study seeks to clarify the structure–reactivity relationship between phenolic acids and proteins under CP treatment and to provide new insights into the development of protein–polyphenol conjugates with improved functional properties for food applications.

## 2 Materials and methods

### 2.1 Materials and chemicals

Hen egg-white ovalbumin (OVA, ≥98% purity) was sourced from Yuanye Bio-Technology Co. Ltd. (Shanghai, China). Chlorogenic acid (CGA, 98%, Lot No. HC021133), caffeic acid (CA, 99%, Lot No. HC230818), and rosmarinic acid (RA, 98%, Lot No. HR222655) were obtained from Herbest Co. Ltd. (Shanxi, China). 5, 5’-Dithiobis (2-nitrobenzoic acid) (DTNB) and 8-anilino-1-naphthalene sulfonic acid (ANS) were purchased from Aladdin Chemicals Co. Ltd. (Shanghai, China). Clinical serum specimens were collected from 11 individuals with confirmed egg allergy diagnoses at the Hunan Provincial Maternal and Child Health Care Hospital (S Table 1) (Approval No. HS-2025-12-010). Folin-Ciocalteu reagent was sourced from Bomei Bio-Technology Co. Ltd. (Hefei, China). Pepsin (≥2500 U/mg) and trypsin (≥1500 U/mg) were supplied by Sigma-Aldrich (St. Louis, MO, USA).

### 2.2 Preparation OVA-RDPAs conjugates by CP treatment

OVA–RDPAs conjugates were prepared according our previous study (Z.-W. Liu, et al., 2025). RDPAs (CGA, CA, RA) at 0.6 mmol/L were mixed with OVA (2 mg/mL) in deionized water after 2 h stirring. Aliquots (5 mL) were exposed to dielectric barrier discharge plasma device (Nanjing Suman Electronic, Co., Ltd., China). After 45 s treatment with (40 k V, 13 k Hz) dielectric barrier discharge cold plasma (CP), the samples were collected and refrigerated at 4 °C for 6 h. Subsequently, the samples were dialyzed (MWCO 8000–14,000 Da) at 4 °C for 48 h to completely remove the free polyphenols that were not combined with OVA. After dialysis, the samples were freeze-dried, and stored in a refrigerator at 4 °C for further analysis. Conjugates were designated OVA-CGA, OVA-CA, and OVA-RA; controls omitted CP treatment.

### 2.3 Total RDPAs determination

Bound phenolic content was determined by Folin-Ciocalteu colorimetry according to Tian et al. (2023). Samples (1 mg/mL in deionized water) were mixed with Folin-Ciocalteu reagent (0.5 mL, 0.2 mol/L), incubated in darkness for 5 min, and then treated with sodium carbonate solution (0.4 mL, 75 mg/mL). Absorbance was measured at 760 nm; phenolic acid content was calculated from standard curves (CGA, CA, RA; S Table 2).

### 2.4 SDS-PAGE analysis

Protein profiles were analyzed by SDS-PAGE according to Song et al. (2024). Samples (1 mg/mL) were mixed with reducing or non-reducing loading buffer (1:4 and 1:3, *v/v*, respectively), with reducing samples heated at 90 °C for 10 min. Aliquots (8 μL sample + 2 μL marker) were loaded and run at 75 V for 2 h. Bands were visualized by Coomassie Brilliant Blue R-250 staining (2 h) and destained with deionized water (4 h).

### 2.5 Particle size and ζ-potential analysis

The average particle size, particle size distribution and ζ-potential of OVA and its conjugates were measured by a Zetasizer Nanosystem (ZETASIZER NANO ZS, Malvern Instruments Ltd., Worcestershire, UK) at 25 °C (Wang et al., 2025).

### 2.6 SEM analysis

Prior to imaging, lyophilized specimens were affixed to aluminum stubs with conductive adhesive and rendered conductive by gold sputter-coating (approximately 10 nm). Surface microstructural features were subsequently visualized on a JSM-6380LV scanning electron microscope (JEOL, Japan) at an accelerating voltage of 10 kV (Yang et al., 2024).

### 2.7 Structural analysis of OVA-RDPAs conjugates

#### 2.7.1 CD analysis

Secondary structure was characterized by CD spectroscopy according to Yang et al. (2024). Protein solutions (0.1 mg/mL in deionized water) were scanned from 190 to 260 nm at 100 nm/min (1.0 nm interval, 1.00 nm bandwidth). Secondary structure fractions (α-helix, β-sheet, β-turn, random coil) were deconvoluted using CDNN software.

#### 2.7.2 UV–vis absorption analysis

UV–vis absorption spectra were recorded on a UV1901G spectrophotometer (Shanghai Yoke Instrument Co., Ltd., China) scanning from 190 to 400 nm. Samples were dissolved in deionized water at 1 mg/mL to evaluate structural changes induced by covalent grafting with CGA, CA, and RA (Wang et al., 2025).

#### 2.7.3 Intrinsic fluorescence spectroscopy analysis

Intrinsic fluorescence was measured according to Zheng et al. (2023). Protein samples (1 mg/mL) were excited at 280 nm with emission recorded across 300–400 nm (5 nm slit widths).

#### 2.7.4 Surface hydrophobicity analysis

Surface hydrophobicity was assessed by ANS fluorescent probe binding according to Yang et al. (2026). Protein solutions (1 mg/mL) were mixed with ANS reagent (50 μL), equilibrated in darkness for 15 min, and then measured with excitation at 390 nm and emission collected from 400 to 600 nm (2.5 nm slit width).

### 2.8 LC-MS/MS analysis

Covalent modification sites were characterized by nano-LC-MS/MS according to the method of Wiśniewski et al. (2009), with modifications. Conjugate samples were digested with trypsin using filter-aided sample preparation (FASP). Briefly, proteins were reduced with 10 mM DTT, alkylated with 50 mM iodoacetamide, and washed with UA buffer (8 M urea, 150 mM Tris-HCl, pH 8.0) and 50 mM NH_4_HCO_3_ in 10 kDa ultrafiltration units. Proteins were then digested with trypsin at 37 °C for 16 h, and the resulting peptides were collected, desalted, lyophilized, and reconstituted in 0.1% formic acid. Peptides were fractionated on C18 trap and analytical columns (250 nL/min) and separated using a binary gradient (4–50% B over 50 min, then 100% over 4 min; B = 0.1% formic acid/84% acetonitrile). Data-dependent acquisition collected ten most intense fragments per survey scan. Raw files were processed in MaxQuant (v2.1.1.1) against OVA UniProt in variable-modification mode (FDR ≤ 0.01).

### 2.9 Functional properties of OVA- RDPAs conjugates

#### 2.9.1 Antioxidant activity

ABTS radical cation (ABTS•+) scavenging capacity was assessed using a modified method of Shang et al. (2024). The reagent was prepared by dissolving 10 mg ABTS and 1.7219 mg potassium persulfate in 2.6 mL deionized water and oxidizing overnight in darkness; stock was then diluted to A_734_ = 0.75 ± 0.02 before use. Protein samples (1 mg/mL in phosphate buffer; 1 mL) were combined with 5 mL working reagent and incubated in darkness for 30 min. Residual was recorded against a buffer blank. Percent radical inhibition was calculated per Eq. (1):

(1) $$\text{ABTS Free Radical Scavenging Activity}\;\left(\%\right)=\frac{A_{0}-A_{1}}{A_{0}}\times 100$$

where A_0_ is the absorbance of the blank control and A_1_ is the absorbance of the sample. The Trolox calibration curve was employed to calculate the ABTS• + scavenging activity (S table 3).

DPPH• scavenging was measured as follows: DPPH dissolved in methanol (0.2 mM) was combined with equal volume (2 mL) of protein solution (1 mg/mL in phosphate buffer) and the reaction maintained in darkness for 30 min; absorbance at 517 nm was recorded against a PBS-methanol blank. Scavenging activity was computed per Eq. (2):

(2) $$\text{DPPH Free Radical Scavenging Activity}\;\left(\%\right)=\frac{A_{b}-A_{s}}{A_{b}}\times 100$$

where A_b_ is the absorbance of the blank control and A_s_ the absorbance of the sample. The Trolox calibration curve was employed to calculate the DPPH• scavenging rate (S table 3).

#### 2.9.2 Emulsifying properties

EAI (m^2^/g) and ESI (min) were quantified using a turbidimetric method adapted from Zheng et al. (2023). An oil-in-water emulsion was formed by homogenization of 6 mL protein solution (1 mg/mL) with 2 mL soybean oil at 12,000 rpm for 2 min. A 50 μL aliquot was immediately dispersed into 4 mL of 0.1% (*w/v*) SDS solution and turbidity at 500 nm recorded at *t* = 0 and *t* = 10 min. EAI and ESI were derived using the equations:

(3) $$\;\mathrm{EAI}\;\left(m^{2}/g\right)=\frac{2\times 2.303\times A_{0}\times D}{c\times \left(1-\phi \right)\times 10000}$$

(4) $$\;\mathrm{ESI}\;\left(\min\right)=\frac{A_{0}}{A_{10}}\times 100\%$$

where 2.303 is the conversion factor between absorbance and turbidity, A_0_ and A_10_ are the absorbance values of the emulsion measured at 0 and 10 min, respectively, D is the dilution factor (4000/50 = 80), c is the protein concentration in the aqueous phase (0.001 g/mL), and ϕ is the oil phase volume fraction (0.25).

### 2.10 *In vitro* digestibility analysis

#### 2.10.1 *In vitro* simulated gastrointestinal digestion

*In vitro* gastrointestinal digestion was conducted following a two-phase sequential protocol based on Z.-W. Liu, et al. (2025). SGF and SIF were formulated as described therein. For the gastric phase, protein solution (1 mg/mL; 10 mL) was mixed 1:1 (*v/v*) with SGF; pepsin (2500 U/mg) was added to yield 2000 U/mL final activity. This corresponds to an enzyme-to-substrate ratio of 4000 U/mg protein, within the INFOGEST recommended range of 2000–4000 U/mg (Brodkorb et al., 2019). Supplemented with 6.5 μL of 0.3 mol/L CaCl_2_, and pH established at 3.0 with 1 mol/L HCl. Gastric hydrolysis proceeded in a thermostated shaker at 37 °C, 60 rpm for 2 h, after which pH was raised to 7.0 with 1 mol/L NaOH to halt peptic activity.

For intestinal digestion, the neutralized gastric digest was combined 1:1 (*v/v*) with SIF containing trypsin (final activity 100 U/mL), bile salts (0.16 mol/L), and CaCl_2_ (20 μL of 0.3 mol/L; pH 7.0 pre-adjusted). Digestion proceeded at 37 °C, 60 rpm for 2 h, then enzymatic activity was quenched by rapid cooling in an ice bath (0–4 °C) for 30 min. All digesta were stored at 4 °C prior to analysis.

#### 2.10.2 Protein digestibility analysis

Protein digestibility was determined by TCA-based precipitation of unhydrolyzed protein following Z.-W. Liu, et al. (2025). Equal volumes (1.5 mL) of digested or undigested sample and 10% (*w/v*) TCA were combined; after overnight precipitation at 4 °C, the mixture was centrifuged at 10,000 rpm for 20 min. Pellets were dissolved in 1 mL of 1 mol/L NaOH and protein quantified by Bradford assay. Digestibility was calculated per Eq. (5):

(5) $$\text{Digestibility}\;\left(\%\right)=\frac{C_{0}-C_{T}}{C_{0}}\times 100$$

where C_0_ and C_T_ represent the concentrations of the total protein and TCA-precipitated protein, respectively. A water-only group was used as the blank control.

### 2.11 Antigenicity analysis

IgE-binding capacity was assessed by indirect ELISA according to Z.-W. Liu, et al. (2025). Samples (400 μg/mL in 50 mM carbonate buffer, pH 9.6) were adsorbed onto microplates (100 μL/well, 4 °C overnight), blocked with 5% skimmed milk (37 °C, 1 h), then incubated with human atopic serum (1:2 dilution, primary, 37 °C overnight), HRP-conjugated goat anti-human IgE (1:5000, secondary, 37 °C, 1 h), and TMB substrate (100 μL/well, 37 °C, 40 min). Reaction was stopped with 2 mol/L H_2_SO_4_ (50 μL/well) and absorbance measured at 450 nm. IgE-binding capacity was calculated per Eq. (6):

(6) $$\mathrm{IgE}\;\text{binding capacity}=\frac{A_{0}-A}{A_{0}-A_{b}}\;$$

where A_0_ and A is the absorbance of OVA and OVA- RDPAs conjugates, respectively, A_b_ is the absorbance of blank control.

### 2.12 Statistical analysis

All experimental determinations were independently replicated three times; data are reported as mean ± standard deviation (*n* = 3). Statistical evaluations were conducted using IBM SPSS Statistics 27.0 (SPSS Inc., USA). All Figures were generated using Origin 2024 software (Origin Lab Co., USA).

## 3 Results and discussion

### 3.1 Covalent binding efficiency of RDPAs in OVA-RDPAs conjugates

**Fig. 1:**
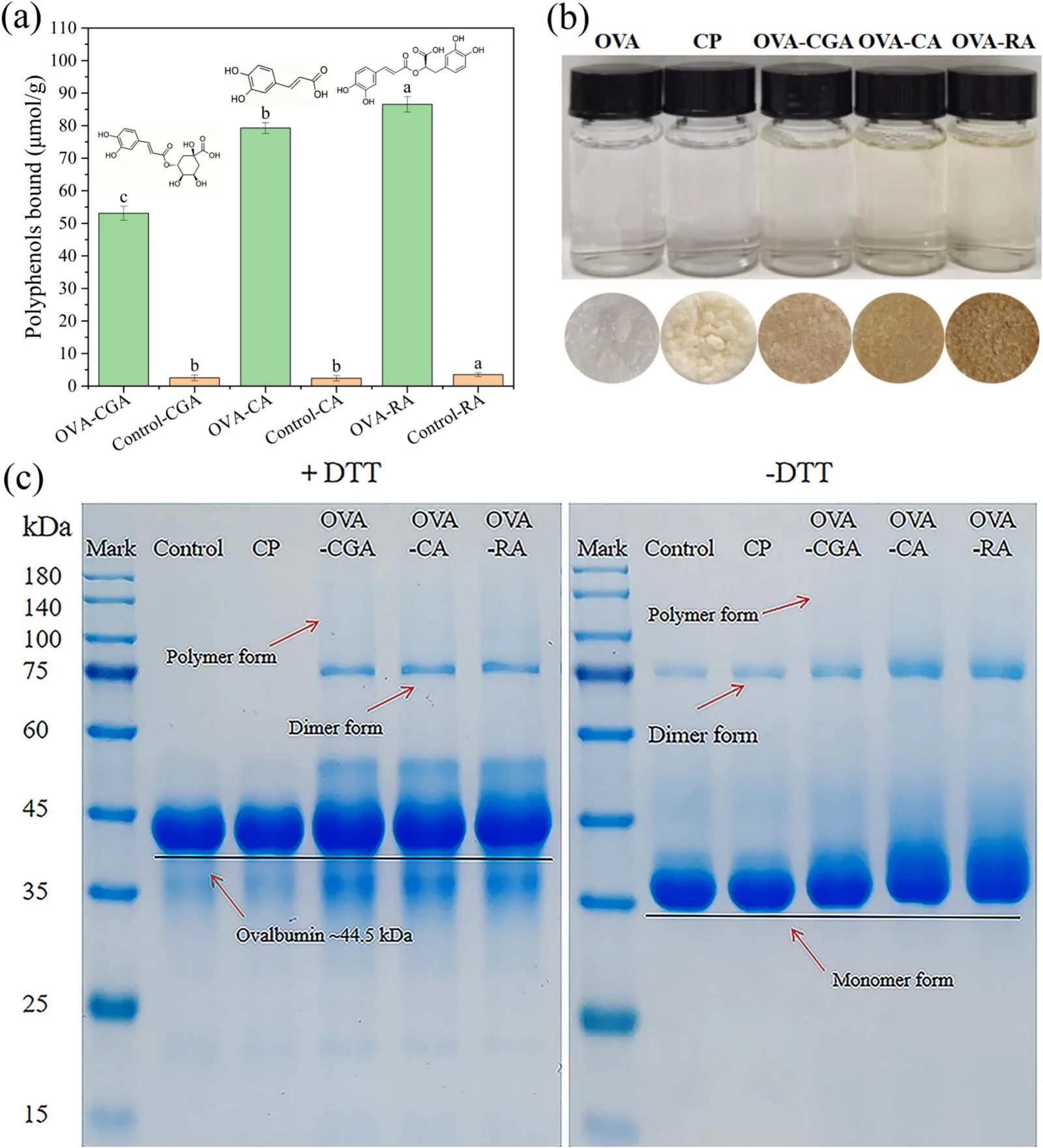
(a) The content of OVA-RDPAs conjugates; (b) The visual appearance of OVA-RDPAs conjugates; (c) The protein pattern of OVA and OVA-RDPAs conjugates analyzed by SDS-PAGE.

To quantitatively evaluate the extent of CP-mediated covalent conjugation between OVA and RDPAs, the number of phenolic compounds grafted onto the resulting OVA–RDPAs conjugates were determined. As shown in Fig. 1a, 45 s of CP treatment markedly increased the incorporated RDPAs content in all three conjugates compared to the untreated control, which remained below 4 μmol/g. Among them, RA exhibited the highest grafting level, reaching 86.55 ± 2.39 μmol/g, followed by CA at 79.27 ± 1.62 μmol/g, whereas CGA showed the lowest incorporation at 53.07 ± 2.16 μmol/g. These results demonstrate that CP treatment effectively promotes covalent coupling between OVA and RDPAs. A similar CP-induced improvement in polyphenol incorporation has been reported by Song et al. (2024), who obtained phenolic contents of 61.54 ± 1.21, 68.32 ± 1.41, and 176.63 ± 1.83 μmol/g for OVA–EC, OVA–EGC, and OVA–EGCG conjugates, respectively. In the present study, the observed differences in conjugation efficiency among the RDPAs can be mainly ascribed to their molecular architecture, particularly the number and spatial arrangement of phenolic hydroxyl groups (Sun et al., 2022). RA possesses four phenolic hydroxyl groups evenly distributed on two terminal aromatic rings, providing a greater density of accessible reactive sites for covalent linkage than CGA and CA, both of which contain only two phenolic hydroxyl group located on a single benzene ring. The lower binding capacity of CGA relative to CA may be associated with steric hindrance caused by the quinic acid-derived cyclohexane-type polyhydroxy carboxylic acid moiety. Moreover, the aliphatic hydroxyl groups in this moiety are likely less involved in covalent conjugation (Wang et al., 2025). From a mechanistic perspective, the formation of covalent conjugates between OVA and RDPAs likely proceeds through reactive intermediates, including semiquinone radicals and rearranged quinone species. These intermediates are produced by the oxidation of aromatic phenolic hydroxyl groups to carbonyl-containing structures, accompanied by the removal of two hydrogen atoms (Yang et al., 2026). This reaction pathway is further supported by the pronounced browning observed in the OVA–RDPAs conjugates (Fig. 1b). Overall, RDPAs with more phenolic hydroxyl groups exhibit higher chemical reactivity and, consequently, a greater tendency to form covalent bonds with OVA.

### 3.2 Protein profile of OVA and its RDPAs conjugates

The formation and molecular weights of the OVA–RDPAs conjugates were confirmed by SDS–PAGE under both reducing (+DTT) and non-reducing (−DTT) conditions. As illustrated in Fig. 1c, the control sample under +DTT displayed a clear band at approximately 45 kDa, corresponding to native OVA. In contrast, all OVA–RDPAs conjugates exhibited obviously upward band shifts along with pronounced smearing, indicating the formation of higher molecular weight species and increased molecular heterogeneity. Moreover, bands corresponding to dimers of OVA (∼90 kDa) and its polymeric aggregates (>140 kDa) were also observed in the conjugate samples. These results were consistent with the phenolic binding equivalent data presented in Section 3.1 and further confirmed that CP treatment effectively induced the covalent grafting of CGA, CA, and RA onto OVA. The appearance of dimeric and polymeric bands also indicated that the RDPAs mediated intermolecular cross-linking between two or more OVA molecules. A similar pattern was observed under –DTT (Fig. 1c), where the major monomeric bands of the OVA–RDPAs conjugate also exhibited an upward shift and pronounced tailing. The stronger dimeric bands under –DTT conditions may be partially associated with disulfide bond-stabilized OVA dimers. These results are consistent with our previous studies (Song et al., 2024) and provide further evidence that CP treatment effectively induced covalent conjugation between OVA and RDPAs**.**

### 3.3 The change in secondary structure of OVA after covalent conjugation with RDPAs

**Fig. 2:**
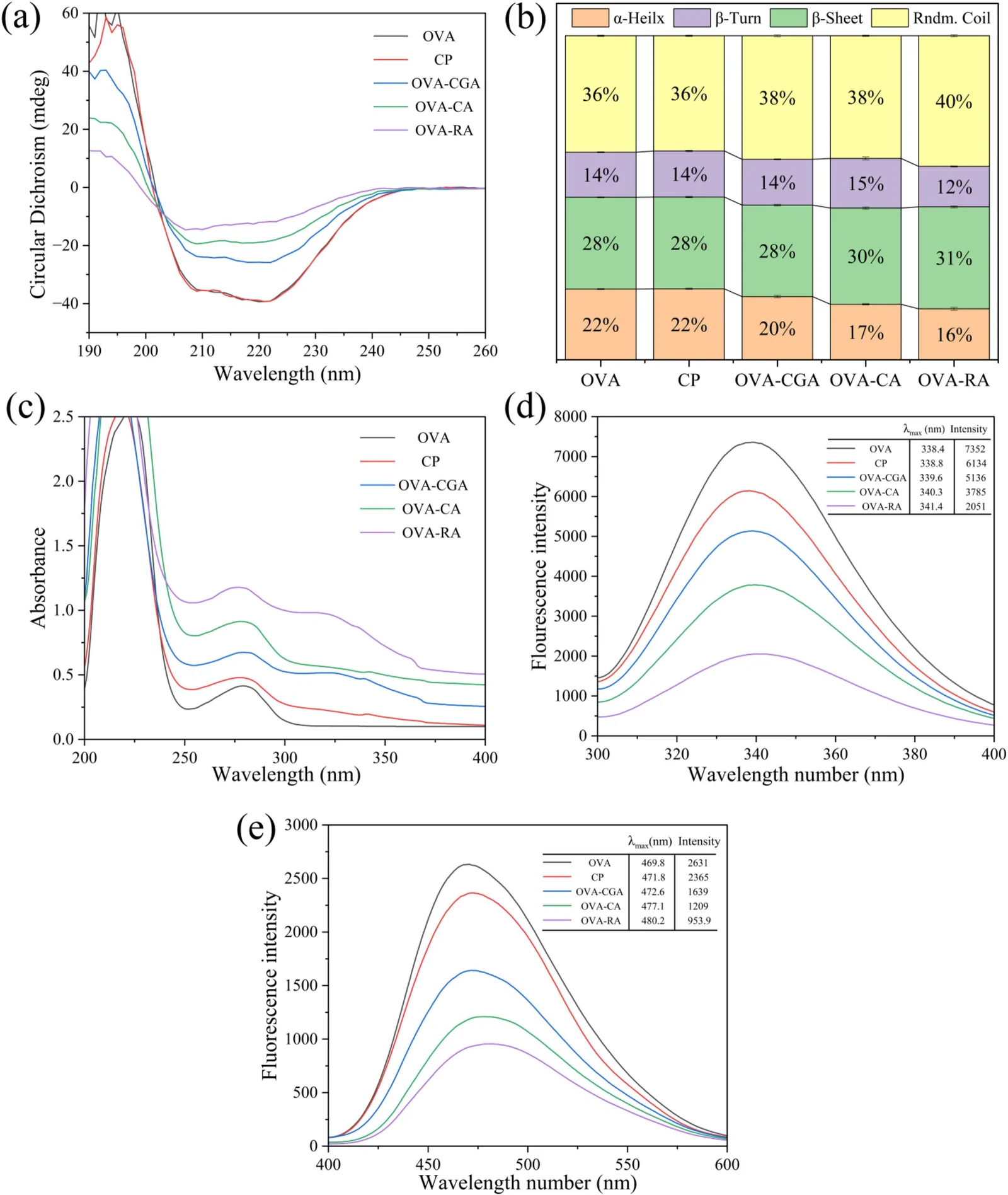
Structural alteration of OVA after covalently conjugated with RDPAs. (a) CD spectra; (b) The content of secondary structure; (c) UV–*vis* spectrum; (d) Intrinsic fluorescence emission spectra; (e) Surface hydrophobicity.

The changes in OVA secondary structures induced by RDPAs were analyzed *via* far-UV circular dichroism (CD) spectroscopy. The dominant negative band of native OVA in the 210–220 nm wavelength range was observed (Fig. 2a). With increased RDPAs grafting amount, the band intensity decreased, implying that the original helical structure was disrupted. The α-helix, β-sheet, β-turn, and random coil contents of native OVA were 22%, 28%, 14%, and 36%, respectively, according to CDNN software (Fig. 2b). There were negligible alterations in the secondary structure of OVA simply treated by CP. Thus, the alterations in the secondary structures of OVA–RDPAs conjugates resulted from the attached polyphenols rather than plasma oxidation. The α-helix and β-turn contents declined while the β-sheet and random coil contents increased as the grafting amount of RDPAs increased. The most notable alteration in secondary structure was observed in OVA–RA; the α-helix decreased from 22% to 16%, the β-turn decreased from 14% to approximately 12%, the β-sheet increased to approximately 31%, and the random coil increased to approximately 40%. The alkaline- and free radical-prepared OVA–RA conjugates exhibited similar changes in OVA secondary structure upon covalent RDPAs conjugation (Shang et al., 2024). Thus, RDPA conjugation can disrupt the native structure of OVA regardless of the grafting method.

### 3.4 UV–vis, intrinsic fluorescence and surface hydrophobic of OVA and its RDPAs conjugates

UV–vis spectroscopy, intrinsic fluorescence spectroscopy, and surface hydrophobicity measurements characterized conformational changes in OVA induced by RDPAs grafting. Native OVA, CP-treated OVA, and all OVA-RDPAs conjugates showed a major absorption peak near 280 nm (Fig. 2c). After RDPAs grafting, absorbance intensity increased slightly, with a blue shift in maximum absorption wavelength (λ_max_) from 279.8 nm (native OVA) to 279.2 nm (OVA-CGA), 278.2 nm (OVA-CA), and 276.8 nm (OVA-RA). This shift indicates that the microenvironment of aromatic residues, particularly Trp and Tyr, changed after conjugation. Zheng et al. (2023) reported similar results for OVA grafted with EGCG *via* ultrasound-assisted free radical methods. The increased absorbance reflects exposure of hydrophobic groups. The blue shift suggests enhanced polarity around these groups and greater conjugation between OVA and RDPAs. OVA-RA additionally showed a new absorption band around 340 nm, likely from RA oxidation during conjugation (Wang et al., 2025).

Intrinsic tryptophan fluorescence (Fig. 2d) revealed progressive quenching and bathochromic emission shifts with increasing RDPAs grafting. FI_max_ declined from 7352 (native OVA) to 5136, 3785, and 2051 for OVA–CGA, OVA–CA, and OVA–RA respectively, while λ_max_ red-shifted from 338.4 nm to 339.6, 340.3, and 341.4 nm in the same rank order (Fig. 2d). Quenching is attributable to inner-filter effects from covalently attached polyphenol chromophores and to relocation of Trp residues to polar solvent-exposed environments upon structural opening (Song et al., 2024). A parallel fluorescence response was documented for EGCG-grafted OVA prepared by sonochemical radical activation (Zheng et al., 2023). Convergent CD, UV–vis, and fluorescence evidence collectively confirm that RDPAs grafting dismantles the compact globular architecture of OVA.

ANS probe binding, quantifying accessible hydrophobic surface area, paradoxically decreased with increasing RDPA grafting (Fig. 2e), most markedly for OVA–RA. Although this appears to contradict the structural opening evidenced by CD and fluorescence, it is consistently explained by competitive surface occlusion: covalently attached RDPAs molecules physically block the same hydrophobic surface patches that ANS would otherwise occupy, irrespective of the degree of structural disorder. The sequence in which the inhibition of ANS fluorescence occurs (OVA-RA > OVA-CA > OVA-CGA) is identical to the grafting ratio, indicating an increase in ANS binding-site blockage at a higher number of grafted polyphenols. An identical observation has been made in WPI-RA grafted through free radicals (Yang et al., 2026), and earlier was considered to be due to aromatic RDPAs groups blocking ANS from accessing hydrophobic areas (Wei et al., 2015).

### 3.5 Particle size, zeta potential and PDI of OVA and its RDPAs conjugates

**Fig. 3:**
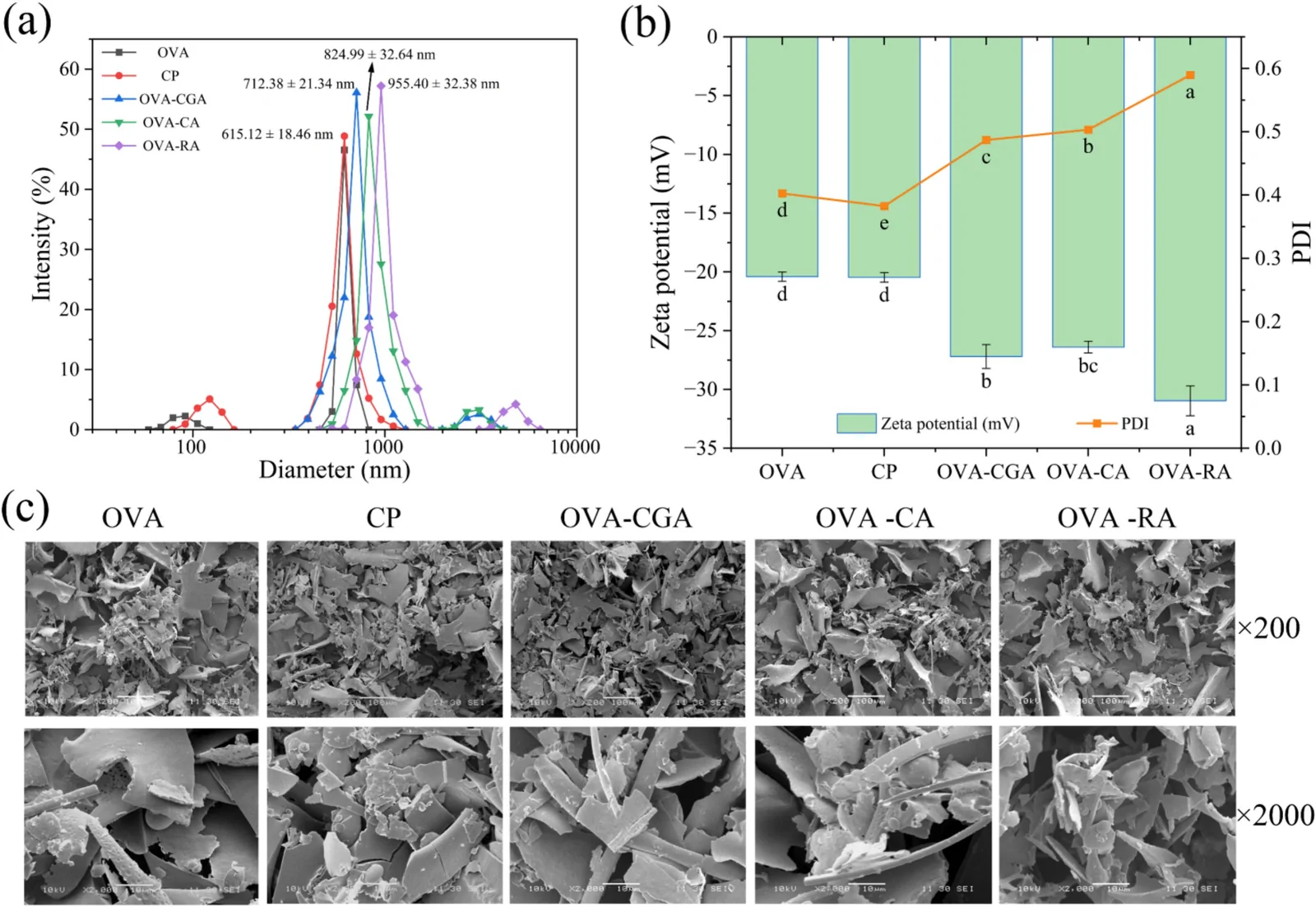
(a) Particle size distribution profiles of OVA and OVA-RDPAs conjugates; (b) Zeta potential of OVA and OVA-RDPAs conjugates; (c) The SEM images of OVA and OVA-RDPAs conjugates, the magnification was set as 200 × (Upper layer) and 2000× (Down layer).

DLS analysis revealed an increment in the size with the addition of the polyphenolic grafted molecules (Fig. 3a). Unconjugated OVA had an average size of 615.12 ± 18.46 nm, and it incremented to 712.38 ± 21.34 nm (OVA-CGA), 824.99 ± 32.64 nm (OVA-CA), and 995.40 ± 32.38 nm (OVA-RA). The increment in the size correlates with the extent of grafting (Section 3.1) and is attributed to the following two mechanisms: an increase in hydrodynamic volume due to RDPAs residue attachment, and hydrophobic surface exposure due to partial unfolding leading to aggregation in solution. Such results have been reported before for OVA crosslinked with phenylaldehydes using ultrasound waves (L. Liu, et al., 2025).

Zeta potential is the core index to characterize the surface charge properties and charge density of colloidal particles. Its determination is of great significance for evaluating the stability of protein and its derivatives and predicting particle behavior (Xing et al., 2025). The zeta potential for native OVA is about −20.4 mV, while there is no appreciable alteration induced by CP alone. Conjugation with RDPAs increased the surface negativity to −26.8 mV (OVA-CGA), −26.4 mV (OVA-CA), and − 30.9 mV (OVA-RA), which is consistent with the introduction of additional ionizable residues such as the carboxylate and phenolate groups of the grafted residues and increased colloidal stability (Wang et al., 2025). The reason may be ascribed to the grafting of negative charge groups such as carboxylic acid group and phenolic hydroxyl of RDPAs on globular surface of OVA (Pham et al., 2019). To the contrast, the PDI of OVA-RDPAs was gradually increased from 0.49 (OVA-CGA), and 0.50 (OVA-CA), to 0.59 (OVA-RA) compared to 0.40 of native OVA. The increase in the PDI of OVA may be attributed to the formation of larger particles with a non-uniform size distribution upon binding with RDPAs. This change is likely due to the formation of dimer and polymer form of OVA cross-linked by RDPAs, which confirmed by the result of SDS-PAGE analysis in section 3.2.

### 3.6 Sem

SEM images showed that there were significant differences in the microstructure of OVA and its RDPAs conjugates (Fig. 3c). Native OVA showed a relatively large, compact layered structure with a smooth surface and clear edges, indicating that it was in a dense aggregation state. CP-treated OVA appeared as irregular, fragmented particles with rough surfaces and a more disordered morphology. In contrast, the microstructure of OVA changed significantly after covalent binding with RDPAs. The OVA-CGA sample still retains part of the sheet structure, but there is obvious fragmentation, and the surface roughness increases. OVA-CA has a looser and more porous structure, with irregular flakes and visible voids, indicating that the original compact structure was destroyed. Among all the conjugates, OVA-RA has the most uneven morphology, with highly fragmented particles and rough porous surfaces. Similarly, Wang et al. (2025) reported that RA grafting OVA can induce protein morphological changes. Similarly, Yang et al. (2026) also observed the WPI-RA complex prepared by free radical method by SEM, and found that grafting RA would lead to more fragmentation of the apparent structure of WPI.

### 3.7 Binding sites of RDPAs in OVA

**Fig. 4:**
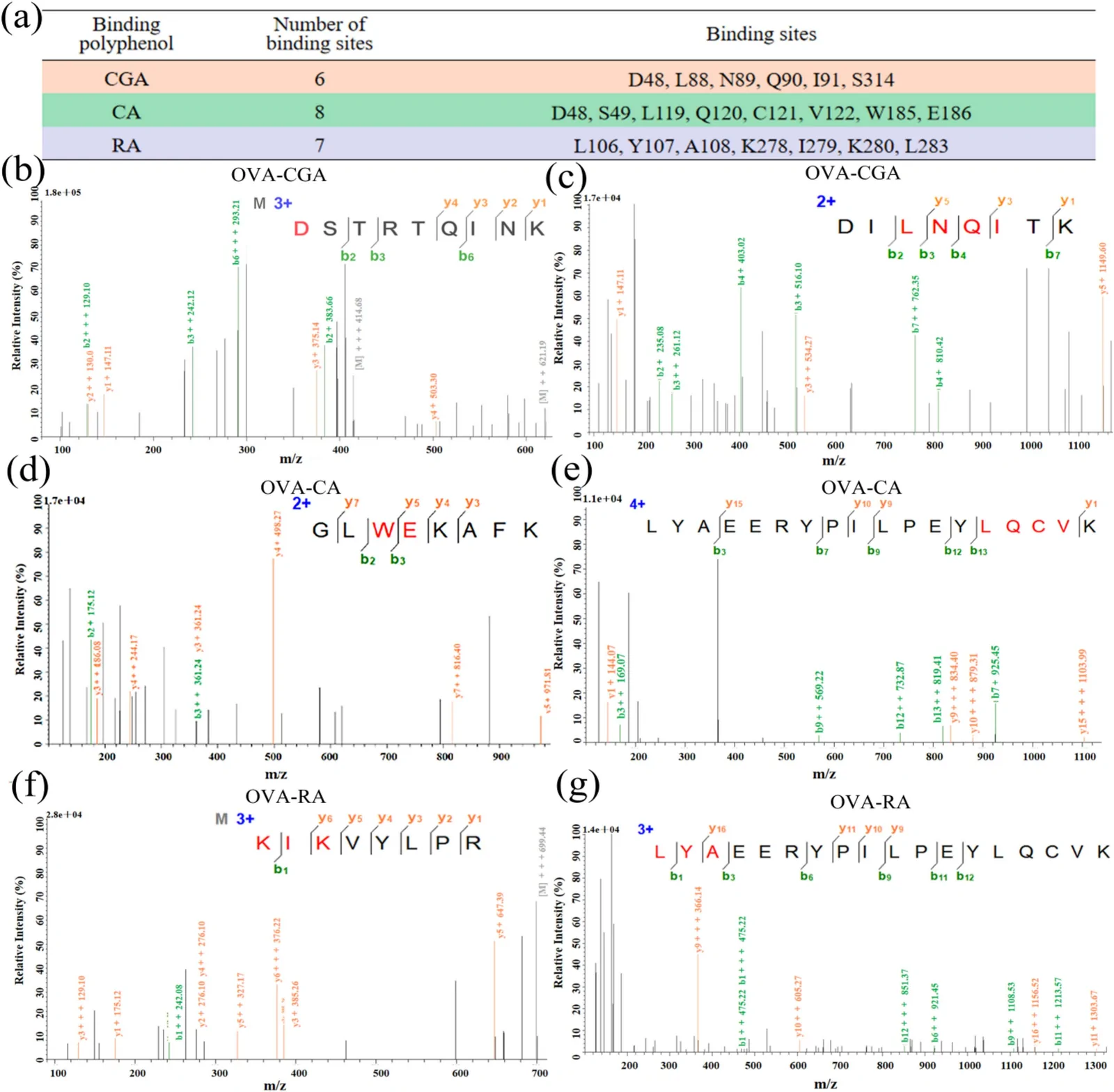
The covalent binding site of RDPAs onto OVA in their conjugates analyzed by LC-MS/MS; (a) The table of LC-MS/MS results; (b) Sequence DSTRTQINK (peptides 48–56) in OVA-CGA; (c) Sequence DILNQITK (peptides 86–93) in OVA-CGA; (d) Sequence GLWEKAFK (peptides 183–190) in OVA-CA; (e) Sequence LYAEERYPILPEYLQCVK (peptides 106–123) in OVA-CA; (f) Sequence KIKVYLPR (peptides 278–285) in OVA-RA; (g) Sequence LYAEERYPILPEYLQCVK (peptides 106–123) in OVA-RA.

The binding sites of RDPAs (CGA, CA, RA) in OVA were further identified by LC-MS/MS. As shown in Fig. 4, CGA, CA, and RA showed different binding regions and numbers of binding sites. In total, 6 binding sites were detected in OVA-CGA, 8 in OVA-CA, and 7 in OVA-RA. Based on the MS2 spectra of the corresponding ions (Fig. 4 b-g), binding sites were identified in D*STRTQINK sequence (peptide 48–56, CGA) and DIL*N*Q*I*TK sequence (peptide 86–93, CGA), GLW*E*KAFK sequence (peptide 183–190, CA), LYAEERYPILPEYL*Q*C*V*KL sequence (peptide 106–123, CA), K*I*K*VYLPR sequence (peptide 278–285, RA) and L*Y*A*EERYPILPEYLQCVK sequence (peptides 106–123, RA). Other binding sites such as Ser314 (CGA), Asp48, Ser49 (CA), and Leu283 (RA) were also found (S Fig. 1). The amino acid immediately preceding the asterisk (*) indicates the modified residue.

Fig. 4c shows the MS2 spectrum of peptide DILNQITK (residues 86–93, CGA), with b and y ions marking cleavage sites. The binding site Leu87 was identified from the mass difference between b2 and b3 ions. Other sites in this sequence (Asn88, Gln89) were similarly identified by ion matching. The MS2 spectrum of peptide GLWEKAFK (residues 183–190, CA) revealed Trp185 bound to CA, again determined by the b2-b3 mass difference. RA binding sites (Ile279, Lys280, Tyr107, Ala108) were detected in peptides KIKVYLPR and LYAEERYPILPEYLQCVK using the same approach. Additional sites included Ser314 (CGA), Asp48 and Ser49 (CA), and Leu283 (RA) (S Fig. 1).

**Fig. 5:**
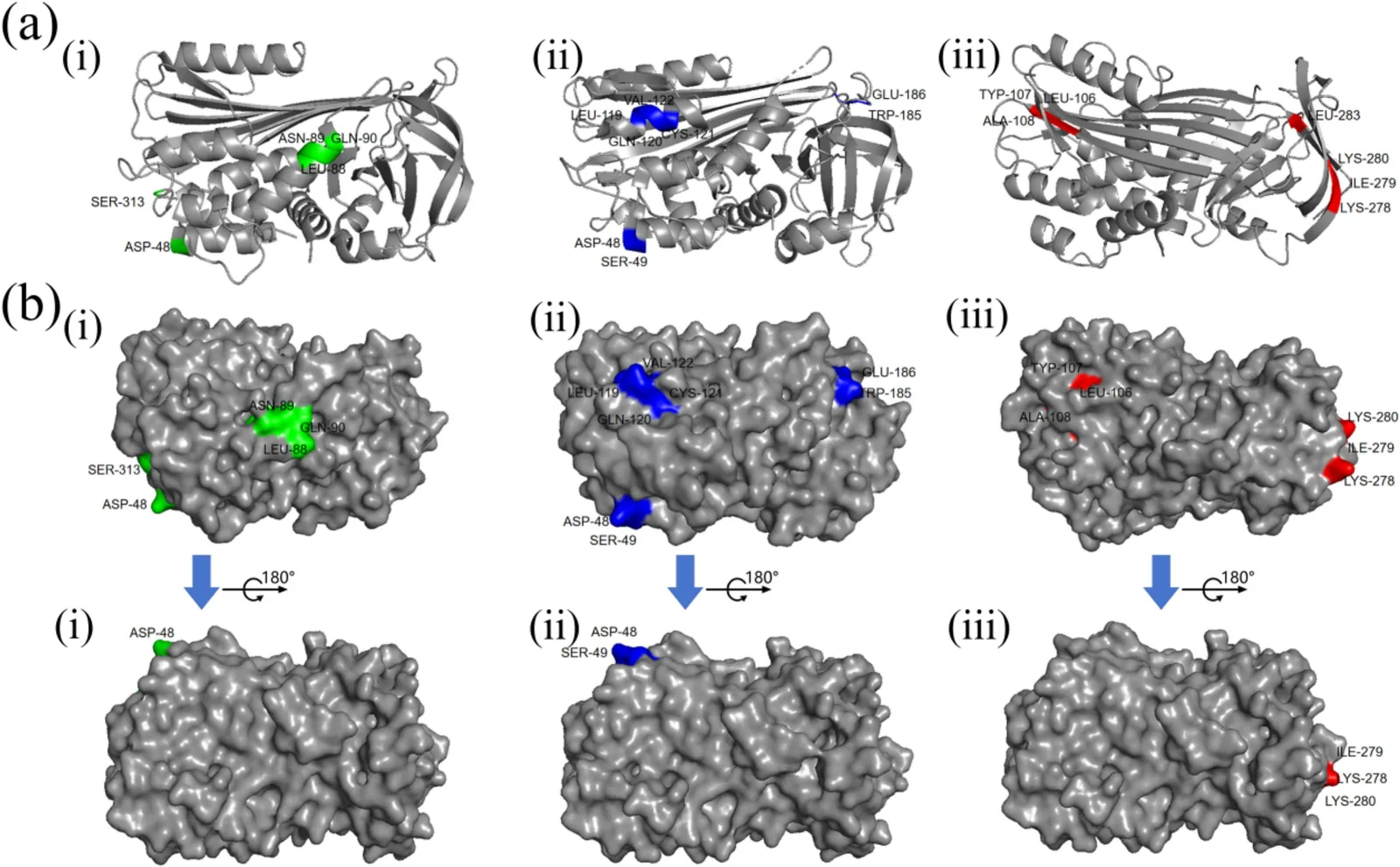
The locations of binding sites of RDPAs onto OVA (PDB ID: 1jti). (a) Schematic representation for the binding sites in the secondary structure OVA. (i) OVA-CGA; (ii) OVA-CA; (iii) OVA-RA; (b) Schematic representation for the binding sites on OVA surface. (i) OVA-CGA; (ii) OVA-CA; (iii) OVA-RA.

According to the results of LC-MS/MS, the binding site characteristics of the RDPAs in OVA are as follows: (1) The peptide LYAEERYPILPEYLQCVK preferentially bound to CA/RA, and a total of seven covalent binding sites were detected in this sequence (Leu106, Tyr107, Ala108, Leu119, Gln120, Cys121, and Val122), with most of these sites located in α-helix and β-sheet structures. (2) Amino acids bearing active nucleophilic side chains (*e.g.*, Lys containing –NH₂ and Ser/Asp containing –OH) and those possessing hydrophobic alkyl side chains (*e.g.*, Ala and Leu) were more prone to covalent conjugation with RDPAs. (3) The majority of the binding sites (Asp48, Ser49, Leu106, Tyr107, Ala108, Leu119, Gln120, Cys121, Val122, Lys278, Ile279, and Lys280) were situated on the surface of the globular OVA molecule (Fig. 5), indicating that spatial accessibility is a critical factor governing the covalent binding of RDPAs.

Consistent with these finding, Shang et al. (2024) declared that RA was primarily grafted onte sulfur-containing amino acids (Met, Cys) and aromatic amino acids (Phe, Tyr) in OVA with free radical method. In contrast, RA preferentially bound to basic or nucleophilic amino acids (His, Arg, Lys) with conventional alkaline method. In the present study, CP-mediated OVA–RDPAs covalent conjugation was not limited to sulfur-containing (Met, Cys) or basic/nucleophilic amino acids (His, Arg, Lys); rather, it also effectively modified hydrophobic residues (Leu, Ile, Ala), aromatic residues (Tyr), and polar residues (Asp, Gln, Ser, *etc.*). Furthermore, CGA, CA, and RA exhibited distinct binding-site profiles on OVA, suggesting that CP not only promotes stable covalent cross-linking between polyphenols and proteins but also preserves the structural specificity associated with different RDPAs molecules.

### 3.8 Functionalities of OVA-RDPAs conjugates

#### 3.8.1 Antioxidant activity

**Fig. 6:**
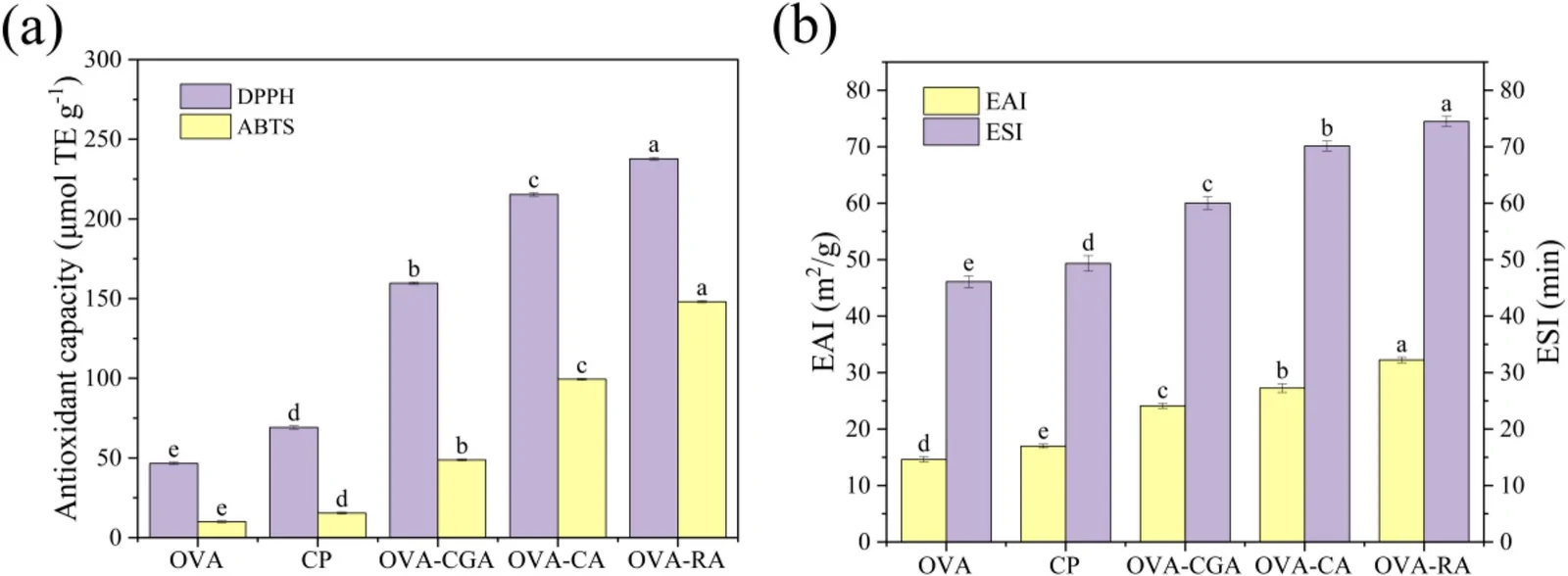
(a) Antioxidant capacity of OVA- RDPAs conjugates; (b) Emulsifying property of OVA- RDPAs conjugates.

CP-mediated RDPAs grafting markedly elevated OVA antioxidant capacity measured by DPPH• and ABTS• + assays (Fig. 6a). DPPH• inhibition rose from 47.93 ± 0.71 μmol TE/g (native OVA) to 160.65 ± 1.02, 215.33 ± 0.94, and 241.40 ± 0.89 μmol TE/g for OVA–CGA, OVA–CA, and OVA–RA, displaying a 3.3- to 5.1-fold gain. ABTS• + scavenging followed the same pattern. OVA–RA reached 150.42 ± 0.66 μmol TE/g *versus* 10.24 ± 0.58 μmol TE/g for native OVA (14.6-fold increase). The improvements are due to the antioxidant nature of covalently bound RDPAs molecules as the ortho di-hydroxy group catechols within them release hydrogen or electrons to neutralize free radicals (Y. Wang et al., 2024). The hierarchy is dictated by not only the number of phenolic hydroxyl groups but also the level of molecular modification, similar to the case of OVA-RA and β-LG-RA molecules synthesized under alkaline conditions (Wang et al., 2024; Wang et al., 2025).

#### 3.8.2 Emulsifying properties

Emulsification performance improved substantially upon RDPAs conjugation (Fig. 6b). EAI rose from 14.61 ± 0.41 m^2^/g (native OVA) to 24.11 ± 0.43, 27.26 ± 0.76, and 32.23 ± 0.47 m^2^/g for OVA–CGA, OVA–CA, and OVA–RA respectively. Corresponding ESI values increased from 46.09 ± 1.01 min to 60.03 ± 1.12, 70.13 ± 0.86, and 74.48 ± 0.89 min. Both factors served as indicators of grafting level by RDPAs, thereby highlighting an apparent relationship between structure and function. There are three possible factors that could be responsible for this: the exposure of the buried hydrophobic sequences through RDPA leads to the increased amphiphilicity of molecules; the high negative surface charge facilitates greater electrostatic repulsion between droplets; and the RDPA-crosslinked oligomers improve cohesion at the film interface (Chen et al., 2018; Zheng et al., 2023).

### 3.9 Allergenicity of OVA-RDPAs conjugates

**Fig. 7:**
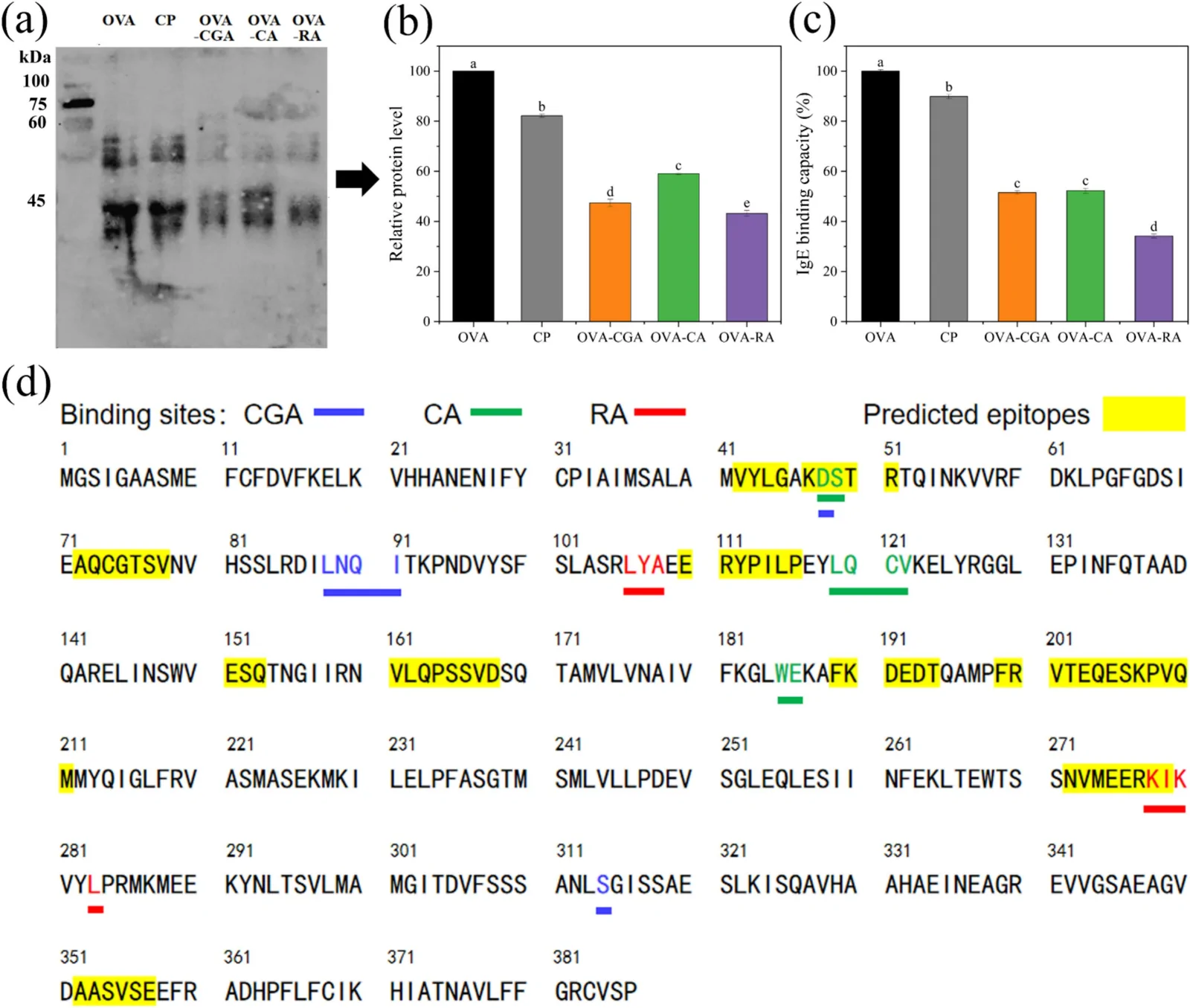
(a) The IgE-binding capacity of OVA-RDPAs conjugates analyzed by WB; (b) Semi-quantitative analysis (based on WB) by Image J; (c) The IgE-binding capacity of OVA-RDPAs conjugates analyzed by ELISA; (d) The location of predicted linear epitopes and binding sites of OVA. The linear epitopes are predicted by DNAStar, Immunomedicine Group, Bepi Pred, Bcepred, and ABCpred.

Immuno-blotting and ELISA techniques were used to investigate the effect of RDPAs conjugation on the reactivity of OVA with IgE antibodies (Fig. 7). In the immuno-blot analysis, the intensities of bands obtained from all RDPAs conjugates were found to be lower compared to those of native OVA. The lowest band intensity was obtained for OVA-RA conjugate, which corresponded to a reduction in allergenicity of 43.23 ± 0.89%, 47.41 ± 1.14%, and 58.93 ± 0.93% for OVA-CGA, OVA-CA, and OVA-RA, respectively (Fig. 7b). Indirect ELISA technique also yielded similar results where OVA-RA had the highest reduction in allergenicity, namely 65.81 ± 0.74%, followed by OVA-CGA and OVA-CA with 52.28 ± 1.01% and 51.51 ± 0.77%. Comparison of the RDPAs conjugation sites as determined by LC-MS/MS with the predicted linear IgE-binding epitopes of OVA (Fig. 7d) explained the molecular basis of allergenicity reduction. A number of the sites of modification mapped directly onto the epitope residues: Asp48 and Ser49 of the CGA- and CA-reactive sequences, and Lys278-Lys280 of the RA-reactive sequence. Furthermore, other sites of modification adjacent to the epitopes would provide steric hindrance for binding of IgE even if the epitopes themselves remained unmodified. CP conjugation of RDPAs can reduce the immunogenicity of OVA by both epitope masking and steric hindrance of the surrounding sequence. The latter is similar to the effects of EGCG-OVA conjugates. He et al. (2019) reported that covalent binding of EGCG masked the linear epitopes at Cys74 and Glu347 in OVA, significantly reducing its allergenicity. Moreover, the extensive unfolding observed by CD and fluorescence spectroscopy likely disrupts the native conformational epitopes of OVA, which, together with epitope masking and steric hindrance, contributes to the reduced IgE binding (Wang et al., 2025).

### 3.10 *In vitro* simulated digestibility of OVA-RDPAs conjugates

The digestibility of OVA and its RDPAs conjugates was evaluated by *in vitro* simulated gastrointestinal digestion model. SDS-PAGE analysis displayed that the covalent conjugates with RDPAs enhanced the *in vitro* digestibility of OVA. Results indicated that the digestibility of OVA was increased after RDPAs conjugation, especially for OVA-CA and OVA-RA, with 20.69 ± 0.85%, 25.37 ± 0.56% decline in band intensity of their OVA conjugates during gastric digestion. Subsequent intestinal digestion, tremendous decrease in band intensity of OVA and its OVA conjugates was observed and achieved the lowest for OVA-RA (25.45 ± 0.18%). Similar results were obtained from the digestibility analysis. During gastric digestion, OVA and its RDPAs exhibited a relatively low digestibility. Compared to native OVA with the digestibility 9.51 ± 0.25%, the digestibility OVA-RDPAs conjugates was slightly increased to 10.12 ± 0.27% for OVA-CGA, 10.55 ± 0.64% for OVA-CA, and 11.59 ± 0.33% OVA-RA. After gastrointestinal digestion, the digestibility of OVA and its RDPAs dramatically increased. And the digestibility of native and CP treated OVA increased to 39.63 ± 1.21% and 41.08 ± 2.33%, respectively. Interesting, the digestibility of OVA stepwise increased after conjugated with RDPAs, with the increased trend of RA (74.78 ± 2.13%) > CA (71.86 ± 2.06%) > CGA (68.74 ± 1.34%), which in line with the binding amount of RDPAs determined in section 3.1. Those results indicated that RDPAs, particularly RA, effectively promoted the digestibility of OVA under *in vitro* simulated digestion conditions. This finding consistent with that of Sun et al. (2022), who reported that covalent grafted by polyphenols induced structural changes of egg white proteins, exposing aromatic hydrophobic amino acids and other functional groups, thereby increasing enzyme-accessible hydrolysis sites and enhancing its *in vitro* digestibility. And this increase in new exposure sites may outweigh the loss of tryptase cleavage at the modified lysine residues. Similarly, He et al. (2019) also observed a positive correlation between OVA and EGCG binding efficiency, the extent of protein conformational changes, and the improvement in digestibility of OVA-EGCG conjugates.

### 3.11 Digesta allergenicity after *in vitro* digestibility of OVA-RDPAs

**Fig. 8:**
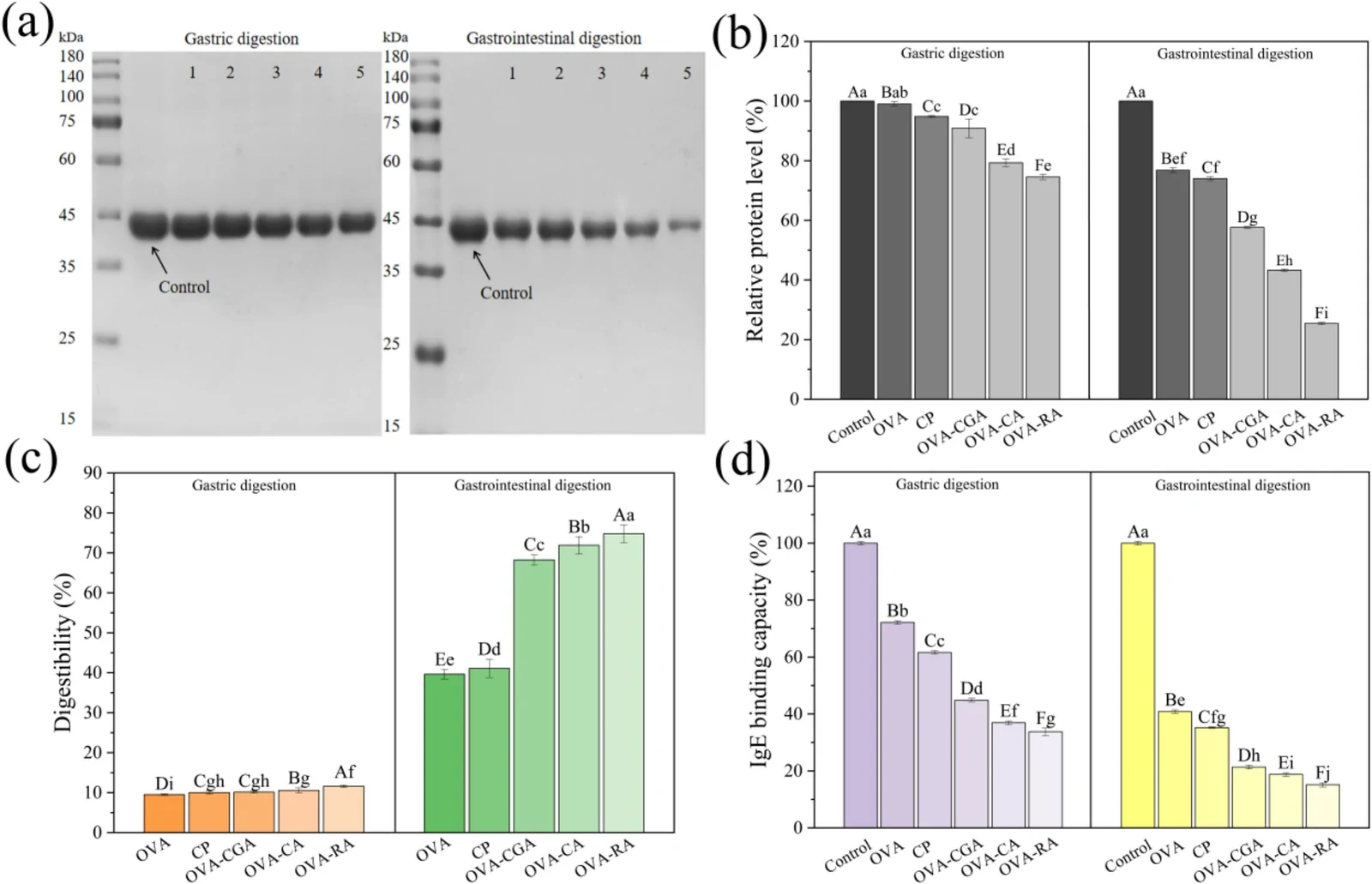
The digestibility and change in IgE-binding capacity of OVA-RDPAs conjugates during *In vitro* simulated gastrointestinal digestion. (a) Digestibility analyzed by SDS-PAGE; (b) Semi-quantitative analysis (based on SDS-PAGE) by Image J; (c) Digestibility analyzed by TCA; (d) Changes in IgE-binding capacity after gastric digestion and gastrointestinal digestion. ANOVA was used, different lowercase letters indicate significant differences within the gastric or intestinal digestion phase, while different uppercase letters indicate significant differences among all samples (*P* < 0.05).

The allergenicity analysis of *in vitro* simulated gastrointestinal digesta revealed that covalent conjugation with RDPAs significantly reduced the immunoreactivity of OVA during *in vitro* simulated gastrointestinal digestion (Fig. 8d). After gastric digestion, native OVA still retained IgE-binding capacity of 72.15 ± 0.66%. Notably, after conjugated with RDPAs, the IgE-binding capacity of OVA-RA declined to 33.76 ± 1.29%, significantly outperforming its OVA-CGA and OVA-CA counterpart (44.87 ± 0.68% and 36.94 ± 0.72%, respectively). Upon subsequent intestinal digestion, IgE-binding capacity of native OVA was further reduced to 40.69 ± 0.61%. OVA-RA conjugates still maintained a relatively lowest IgE-binding capacity (15.15 ± 0.73%), followed by OVA-CGA and OVA-CA conjugates, with IgE-binding capacity of 21.35 ± 0.66% and 18.71 ± 0.68%, respectively. In conclusion, covalent conjugation of RDPAs effectively attenuated the immunoreactivity of OVA during *in vitro* simulated gastrointestinal digestion, especially after gastric digestion.

During the gastric digestion phase, proteolysis was limited, thus, the reduction in allergenicity was mainly attributed to direct masking and steric shielding of IgE-binding linear epitopes by RDPAs. As described in Section 3.9, certain RDPAs binding sites were located within or adjacent to predicted linear epitope regions, thereby hindering antibody recognition. Meanwhile, conformational unfolding induced by covalent conjugation may have disrupted conformational epitopes, further attenuating immunoreactivity (Shang et al., 2024). Owing to their unfolded structures, RDPA conjugates exposed more enzymatic cleavage sites, facilitating preferential degradation of epitope-containing peptides (Tong et al., 2018). Among the conjugates, RA binding involved Lys278 and Lys280, which are trypsin-specific cleavage sites (Sun et al., 2022). RDPAs grafting at these sites may alter the enzymatic cleavage pattern, promote the formation of non-immunogenic short peptides, and accelerate epitope destruction. Because RA contains two catechol moieties and has the highest density of phenolic hydroxyl groups, its binding sites covered three independent regions adjacent to linear epitopes. This broader cross-linking pattern may have resulted in more extensive epitope masking and, consequently, superior allergenicity reduction after gastrointestinal digestion (C. Liu, et al., 2025). In summary, RDPAs covalent conjugation continuously diminished the immunoreactivity of OVA throughout digestion through a synergistic mechanism involving steric epitope masking, conformational epitope disruption, and targeted modification of enzyme cleavage sites, thereby effectively reducing OVA allergenicity.

## 4 Conclusion

This study demonstrated that CP effectively promoted the covalent conjugation of OVA with RDPAs, thereby modulating its structure, functionality, *in vitro* digestibility, and allergenicity in a phenolic acid structure-dependent manner. Among the tested phenolics, RA exhibited the highest grafting efficiency, followed by CA and CGA, underscoring the critical role of hydroxyl-group distribution and steric effects in determining conjugation reactivity. CP-mediated grafting induced pronounced conformational unfolding and intermolecular cross-linking of OVA, as evidenced by spectroscopic and electrophoretic analyses, with the most substantial structural perturbation observed for OVA–RA. These molecular alterations were accompanied by marked improvements in functional performance, particularly antioxidant capacity. More importantly, RDPAs incorporation significantly reduced IgE-binding capacity, with OVA–RA achieving the greatest decrease. In addition, CP- mediated RDPAs conjugation promoted the gastrointestinal digestion of OVA, and the resulting digests retained markedly reduced IgE-binding affinity, highlighting the stability of the hypoallergenic effect after digestion. Collectively, these findings establish CP-mediated RDPAs conjugation as a mild and efficient strategy for tailoring OVA into multifunctional ingredients with enhanced bioactivity and reduced allergenicity. Among the phenolics examined, RA emerged as the most promising modifier, providing a rational basis for the design of value-added and multifunctional egg protein ingredients for food applications.

## CRediT authorship contribution statement

**Zhi-Wei Liu:** Writing – review & editing, Writing – original draft, Supervision, Methodology, Funding acquisition, Data curation, Conceptualization. **Xiang-Xiang Xu:** Writing – review & editing, Formal analysis, Data curation, Conceptualization. **Zi-Jing Yin:** Writing – review & editing, Formal analysis, Data curation. **Waqar Ahmed:** Writing – review & editing, Methodology. **Marwa Ezz El-Din Ibrahim:** Writing – review & editing, Methodology. **Rana Muhammad Aadil:** Writing – review & editing, Supervision, Methodology, Conceptualization. **Hong-Ying Guo:** Writing – review & editing, Supervision, Methodology, Investigation, Data curation, Conceptualization.

## Declaration of competing interest

The authors declare that they have no known competing financial interests or personal relationships that could have appeared to influence the work reported in this paper.

## Data Availability

Data will be made available on request.
