# Supplementary material for: Modulation of structure, functionality, allergenicity and in vitro digestibility of ovalbumin through cold plasma-induced conjugation with rosemary-derived phenolic acids
Source: Food Chem X. 2026 Sep 6;39:104409. doi: 10.1016/j.fochx.2026.104409 (PMC13578954; doi:10.1016/j.fochx.2026.104409)
Supplement: Supplementary file 1 [file mmc1.docx]

**Supplementary material**

S Table 1 Specific IgE levels (kU/L) of 11 patients against egg white

| Patient | Sex | Age (years) | IgE levels (kU/L) |
| --- | --- | --- | --- |
| 1 | Female | 0.7 | 5.22 |
| 2 | Female | 4 | 0.63 |
| 3 | Female | 0.7 | 0.37 |
| 4 | Male | 1 | 1.27 |
| 5 | Female | 2 | 42.24 |
| 6 | Female | 0.8 | 4.77 |
| 7 | Male | 3 | 8.19 |
| 8 | Female | 0.7 | 0.37 |
| 9 | Male | 2 | 1.65 |
| 10 | Male | 4 | 4.46 |
| 11 | Female | 29 | 3.30 |

S Table 2 Standard curves derived from CGA, CA and RA

| Phenolic Acids | standard curves | R^2^ |
| --- | --- | --- |
| CGA | y = 6.7176x + 0.0259 | 0.99 |
| CA | y = 8.3446x + 0.0389 | 0.99 |
| RA | y = 9.7559x + 0.0435 | 0.99 |

Where X indicates the polyphenols content 1mg/mL, and Y denotes the Abs Measured at 760 nm.

S Table 3 The Trolox calibration curve

| Freeradical | Measurement wavelength | Standard curves (R^2^ >0.999). |
| --- | --- | --- |
| ABTS | 517 nm | Y = 26.359X + 0.1489 |
| DPPH | 734 nm | Y = 35.734X - 3.533 |

Where X indicates the equivalent concentration of Trolox in mg/mL, and Y denotes scavenging rate.

S Fig. 1 The covalent binding site of OVA-RDPAs conjugates analyzed by LC-MS/MS; (a) Sequence YNLTSVLMAMFITDVFSSSANLSGISSAESLK (peptides 290 - 321) in OVA-CGA ; (b) Sequence DSTRTQINK (peptides 48 - 56) in OVA-CA ; (c)Sequence DSTRTQINK (peptides 279 - 287) in OVA-RA .
